# The Epitope-Specific Anti-human CD4 Antibody MAX.16H5 and Its Role in Immune Tolerance

**DOI:** 10.3389/fimmu.2019.01035

**Published:** 2019-05-24

**Authors:** Lilly Stahl, Anna Duenkel, Nadja Hilger, Uta Sandy Tretbar, Stephan Fricke

**Affiliations:** ^1^Immune Tolerance Unit, Fraunhofer Institute of Cell Therapy and Immunology, Leipzig, Germany; ^2^Max-Bürger Research Center, Institute for Clinical Immunology, University of Leipzig Medical Center, Leipzig, Germany

**Keywords:** T cell modulation, anti-human CD4 antibody, MAX.16H5, autoimmune disease, graft-vs.-host disease, graft-vs.-leukemia effect

## Abstract

T cell modulation in the clinical background of autoimmune diseases or allogeneic cell and organ transplantations with concurrent preservation of their natural immunological functions (e.g., pathogen defense) is the major obstacle in immunology. An anti-human CD4 antibody (MAX.16H5) was applied intravenously in clinical trials for the treatment of autoimmune diseases (e.g., rheumatoid arthritis) and acute late-onset rejection after transplantation of a renal allograft. The response rates were remarkable and no critical allergic problems or side effects were obtained. During the treatment of autoimmune diseases with the murine MAX.16H5 IgG_1_ antibody its effector mechanisms with effects on lymphocytes, cytokines, laboratory and clinical parameters, adverse effects as well as pharmacodynamics and kinetics were studied in detail. However, as the possibility of developing immune reactions against the murine IgG_1_ Fc-part remains, the murine antibody was chimerized, inheriting CD4-directed variable domains of the MAX.16H5 IgG_1_ connected to a human IgG_4_ backbone. Both antibodies were studied *in vitro* and in specific humanized mouse transplantation models *in vivo* with a new scope. By *ex vivo* incubation of an allogeneic immune cell transplant with MAX.16H5 a new therapy strategy has emerged for the first time enabling both the preservation of the graft-vs.-leukemia (GVL) effect and the permanent suppression of the acute graft-vs.-host disease (aGVHD) without conventional immunosuppression. In this review, we especially focus on experimental data and clinical trials obtained from the treatment of autoimmune diseases with the murine MAX.16H5 IgG_1_ antibody. Insights gained from these trials have paved the way to better understand the effects with the chimerized MAX.16H5 IgG_4_ as novel therapeutic approach in the context of GVHD prevention.

## Introduction

Besides the T cell receptor (TCR) and the CD3 antigen, other molecules are expressed on T cells but are also present on other hematopoietic cells (1). Monoclonal antibodies targeting such antigens (other than the TCR or CD3) can therefore bind to several antigen-expressing cell types. The CD4 molecule is expressed on T cells, monocytes and macrophages and contains four immunoglobulin-like domains (D1–D4) (2). It acts as a co-receptor during antigen presentation and associates with the TCR upon major histocompatibility complex (MHC) binding (1, 2). In the past, several therapeutic strategies using CD4-directed antibodies were investigated for the treatment of several autoimmune diseases [reviewed in Wofsy (3) and Burmester et al. (4)]. In this context, the human CD4^+^ T-cell clone 2C11 was generated (5) for immunization of BALB/c mice to produce the monoclonal anti-human CD4 antibody MAX.16H5 (initial name 30F16H5) (6, 7). Therefore, splenocytes of the immunized mice were fused with X63-Ag8.653 mouse myeloma cells to generate hybridoma cells (6–8). To examine their binding properties, antibody-containing hybridoma supernatants were incubated with CD4^+^ T cells prepared from peripheral blood (PB) which were subsequently analyzed using enzyme-linked immunosorbent assays (ELISA) and cytofluorometric analyses (6). For the development as a therapeutic antibody, MAX.16H5 IgG_1_ was selected because of its high affinity to CD4 (9). In this study, researchers compared 225 different CD4-directed antibodies regarding their CD4 binding properties and kinetics showing that MAX.16H5 IgG_1_ shared some fine specificities with gp120 with regard to the recognition of different mutated CD4 versions (9). At the same time, experiments were performed to obtain information about the binding properties of gp120 and MAX.16H5 IgG_1_ to the CD4 molecule by using peptides (10). The peptide T_b_YIC_b_E_b_VEDQK_Ac_EE was reported to inhibit CD4 binding of both gp120 and MAX.16H5 IgG_1_ (10). During the early years, the antibody was tested in several different assays thereby obtaining solid information not only about antigen-binding properties, but also about antibody-mediated effector mechanisms. Throughout the clinical development of the murine MAX16.H5 IgG_1_, pharmacodynamic and pharmacokinetic data were collected. Upon administration in patients, the mode-of-action of the antibody, the induced CD4 and immunomodulation were studied intensively. Since MAX.16H5 IgG_1_ was applied systemically, clinical data implementing cytokine profiles, acute-phase-reactant evaluation and side effects were obtained and documented. This review summarizes the clinical development of the therapeutic use of MAX.16H5 IgG_1_ for the treatment of autoimmune diseases toward a promising treatment option for hematopoietic stem cell transplantation (HSCT)-related GVHD.

## MAX.16H5 IgG_1_ in the Treatment of Autoimmune Diseases

In total, 47 patients have been treated with the murine wild type antibody MAX.16H5 IgG_1_ (7, 11–24). The individuals suffered from varying diseases or conditions: rheumatoid arthritis (RA), systemic lupus erythematosus (SLE), inflammatory bowel disease (IBD), or acute late-onset rejection after transplantation of a renal allograft (Table 1). Thereby, RA was the most studied disease. Besides the studies focusing on the applicability of MAX.16H5 IgG_1_ as a promising therapeutic antibody format, several studies were published about the use of ^99m^Tc-labeled MAX.16H5 IgG_1_ in RA patients to report the localization/accumulation, the pharmacokinetics, and the elimination process of the antibody (7, 11, 23, 24). A broad variety of parameters was obtained during these clinical trials to evaluate the safety and efficacy of the systemic therapeutic administration of MAX.16H5 IgG_1._ Table 1 provides an overview of the studies performed in humans.

**Table 1:** Results of human studies using MAX.16H5 IgG_1_.

**Underlying disease**	***N***	**MAX.16H5 IgG_**1**_ treatment**	**RR**	**Adverse effects and HAMA development**	**References**
Active, severe RA	6	5/6 patients: 200–300 μg iv (370-550 MBq) ^99m^Tc-mAb 1/6 patients: ≥10 MBq of lymphocytes treated *in vitro* with ^99m^Tc-mAb	n.r.	No adverse effects observed	(7)
Active, severe RA	10	0.3 mg/kg BW [20 mg/day (14)] iv on 7 consecutive days; repeated treatment cycle after 8 weeks (4/10 patients)^a^	9/10^b^	2/10 patients: chills with fever, possibly due to lymphokine release syndrome (13) 2/10 patients: urticaria, 1/2 with severe allergic reaction possibly triggered by keeping a rodent as a pet, patient withdrawn from study (13) Chills, tremor, elevated body temperature, and nausea (15) Systemic side effects correlated with elevated levels of TNF-α, IL-2, and IFN-γ (15) 5/10 patients: HAMA production after 1st treatment cycle → of these 3/4 showed HAMA production after 2nd treatment cycle (16)	(13–16) (19) (17) (18)
Chronic active steroid-resistant or steroid-dependent IBD	3	0.3 mg/kg BW iv on 7 consecutive days^c^	3/3^d^	No adverse effects observed	(20)
Severe acute rejection after renal allograft	11^e^	5/11 patients: 0.6 mg/kg BW iv on 3 consecutive days^e^	3/5^e^	No adverse effects reported	(22)
Intractable severe SLE	1	0.3 mg/kg BW iv on 7 consecutive days^f^	1/1^g^	No adverse effects observed	(12)
Active, severe RA	4	3/4 patients: ≤ 250 μg ^99m^Tc-mAb iv^h^ 1/4 patients: ≥ 10 MBq of lymphocytes treated *in vitro* with ^99m^Tc-mAb^h^	n.r.	No adverse effects observed	(11)
Active, severe RA or healthy control	8	200–300 μg (370–550 MBq) ^99m^Tc-mAb and/or 1 mg (370 MBq) iv polyclonal HIG	n.r.	No adverse effects reported	(23)
Active, severe RA	1	2 mg (810 MBq) ^99m^Tc-anti-CEA IgG_1_ iv, 9 days later 250 μg (910 MBq) ^99m^Tc-MAX.16H5 IgG_1_ iv	n.r.	No adverse effects reported	(24)
Active, severe systemic onset JCAF	2	2 courses of 0.3 mg/kg BW iv on 7 consecutive days (time interval: 8 weeks)^i^	3/3^j^	No side effects after first treatment course 1/2 JCA patients: urticarial rash after first infusion of the second course	(21)
“adult type” RA	1	single course of 0.3 mg/kg BW iv on 7 consecutive days		1/2 JCA patients: fever up to 39.5°C with chills after the first antibody infusion of the second course Further infusions of the second treatment course well tolerated HAMA development detected in both JCA patients after two treatment courses No alterations in organ function, no infections observed either during treatment or during a 6months follow up in JCA patients No side effects reported for third patient with “adult type” RA	

The study references are mentioned, as well as the study population N, the underlying disease of the patients, the treatment conditions with MAX.16H5 IgG_1_, the response rate RR, and—if so—observed adverse effects and HAMA development.

*BW, body weight; CEA, carcinoembryonic antigen; CRP, C-reactive protein; ESR, erythrocyte sedimentation rate; HAMA, human anti-mouse antibody; HIG, human immunoglobulin; IBD, inflammatory bowel disease; inj., injection; iv, intravenous; JCA, juvenile chronic arthritis; mAb, monoclonal antibody; n.r., not reported; NSAID, non-steroidal anti-inflammatory drug; RA, rheumatoid arthritis; RR, response rate; SLE, systemic lupus erythematosus*.

a *NSAID and steroid schedules were not changed during the observation period. “Treatment with slow acting anti-rheumatic agents had been discontinued in all but one patient at least 8 weeks before treatment” (18). Low-dose cyclophosphamide (50 mg/day) was maintained in one patient. Analysis of HAMA production in a 2^nd^ treatment cycle after 6–8 weeks (16)*.

b *“Responders were defined by a reduction of the Ritchie articular index of more than 30% of the initial levels 4 and 8 weeks after treatment or by a decrease of ESR and CRP values of more than 50%” (18). In 3 patients, clinical improvements were only achieved after the second treatment course. One patient withdrew from the study due to an apparently allergic reaction and was excluded from the discussion*.

c *Aside from the antibody treatment, 1.5 g (2 patients) or 3 g (1 patient) mesalazine were given together with 10 mg prednisolone throughout the observation period*.

d *One patient's clinical parameters improved for 3 weeks after the treatment; after 4 weeks he had a mild relapse. The second patient underwent a transient improvement but relapsed after 1 month. The last patient had a complete clinical, endoscopic, and biochemical remission for more than 5 months*.

e *Six other patients with severe acute rejection 1.5–8 years post transplantation received 3 × 1 g methylprednisolone alone without antibody therapy./ Responders to anti-CD4 therapy were characterized by creatinine levels below 50% of maximum increase 4 weeks after rejection treatment*.

f *Prednisolone therapy (50 mg/day) was continued throughout the observation period*.

g *Clinical and laboratory improvements lasted for 4 weeks after the antibody therapy. At this time point, methylprednisolone was given as bolus therapy for 5 days (750 mg daily) resulting in complete remission proven by the (for the first time) negative anti-DNA-antibody titer*.

h *“Four weeks before scintigraphy, conventional anti-inflammatory therapy was stopped whereas ongoing steroid treatment was continued with < 10 mg/d” (11)*.

i *One patient was concomitantly treated with 3 × 25 mg diclofenac, 15 mg prenisone (reduced to 10 mg during the second treatment course), and 2 × 100 mg cyclosporine (solely during the first treatment course) daily, and the other patient with 2 × 250 mg naproxen, 10 mg prednisone (reduced to 7.5 mg during the second treatment course), and 17.5 mg methotrexate daily*.

j *A 50% reduction of the Ritchie index, 65% reduction of the number of swollen joints, and disappearance of morning stiffness as well as a clear improvement of the CRP levels was defined as treatment success. “There were immediate beneficial clinical effects of treatment in one patient, while in the other marked beneficial effects were achieved only by repeated treatment. These effects could not be attributed to longstanding treatment with immunosuppressants” (21). Moreover, concomitant medication could be reduced in both JCA patients after the first treatment course*.

Since it is known that murine antibodies can cause immunological reactions in humans, the administration of the murine MAX.16H5 IgG_1_ was particularly examined. Immunological reactions against the Fc-part of the murine antibody were expected, and in three different studies that administered MAX.16H5 IgG_1_ in the background of RA the production of human anti-mouse antibodies (HAMAs) was investigated (13, 16, 21). Based on the obtained datasets authors concluded that HAMA production followed by MAX.16H5 IgG_1_ administration was rather low and that HAMA activities are directed against specific determinants of the antibody, including anti-idiotypic reactivity (16). Furthermore, it was shown that MAX.16H5 IgG_1_ F(ab)_2_ directed HAMA (IgG) levels did not exceed levels higher than 0.7 mg/l after the first and 1.7 mg/l after the second course of therapy (13). Compared to HAMA activities exceeding 100 mg/l measured in other studies using monoclonal murine antibodies against cancer antigens (25, 26) in immunocompetent patients, the HAMA amounts directed against MAX.16H5 IgG_1_ were rather low, but detectable. Overall, even in studies using the murine IgG_1_ isotype of MAX.16H5, only low HAMA amounts were detected which allowed for further treatment cycles without loss of efficacy (16).

### MAX.16H5 IgG_1_ Mediated Effector Mechanisms

Antibodies can mediate effector mechanisms by both binding the antigen via the Fab domain and binding Fc receptors (FcRs) expressed on effector cells through the Fc part. Mouse IgG_1_ is known to bind two different murine Fc receptors, mFcγRIIb and mFcγRIII (27). The mFcγRIIb is an immunoreceptor tyrosine-based inhibitory motif (ITIM)-carrying receptor, which is highly expressed on murine B cells, granulocytes, macrophages, monocytes and dendritic cells (27). In contrast, mFcγRIII is absent on B cells but highly expressed on monocytes, macrophages, dendritic cells, and granulocytes thereby mediating activating signals via an immunoreceptor tyrosine-based activation motif (ITAM) [reviewed in Bruhns (27)]. Mice produce three different IgG subclasses which do not only differ in their FcR binding specificity but also bear diverse capacity to activate the complement system (27–30). Based on serum bactericidal activity measurements the following hierarchy of murine IgG induced complement activity was proposed: IgG3 > IgG2 > IgG1 (30). It has to be noted that in these assays human serum was used as a source of complement (30).

To this date, no MAX.16H5 IgG_1_ mediated complement-dependent cytotoxicity (CDC) (using rabbit serum as a source of complement) or antibody-dependent cell-mediated cytotoxicity (ADCC) using granulocytes or peripheral blood monocytes as effector cells was obtained in *in vitro* assays (13, 31).

#### Effects on Lymphocytes

In general, the MAX.16H5 IgG_1_ treatment results in a decrease of CD4^+^ cells in RA patients and therefore to an overall reduction of CD3^+^ cells (13, 14). Neither CD8^+^ nor B-cell values were changed in RA patients (13, 14). Immune cells from RA patients treated with MAX.16H5 IgG_1_ showed reduced proliferation to various stimulatory agents 1 h post injection (*p.i*.) (13). However, in four out of nine patients, increased mitogen responses were induced after 8 days, which was indicative for unaltered clinical effects in these patients (13). Blood samples from IBD patients treated with MAX.16H5 IgG_1_ showed a reduced lymphocyte proliferation after stimulation with mitogens and recall antigens (20), too.

Immortalized and interleukin (IL)-2-dependent CD4^+^ T cells revealed reduced mitotic activity (not increased apoptosis) after incubation with MAX.16H5 IgG_1_ or its F(ab')_2_ (32). The same effect was observed with the Fab of MAX.16H5 and gp120 of HIV which could be prevented by high concentrations of IL-2 (32). The authors showed that this effect was connected to a decreased amount of lymphocyte-specific protein tyrosine kinase (Lck) bound to the intracellular domain of CD4 (32).

For further investigation of intracellular signaling pathways, the calcium release after TCR stimulation was examined in peripheral blood mononuclear cells (PBMCs) of RA patients or healthy donors treated with MAX.16H5 IgG_1_ (33, 34). In one study, samples from healthy donors showed reduced intracellular calcium levels after MAX.16H5 IgG_1_ incubation and TCR stimulation *in vitro*, but only if MAX.16H5 IgG_1_ was still bound to the CD4 molecule (33). In a second study, the intracellular calcium concentration did not increase after solely incubation with MAX.16H5 IgG_1_ (34). Only after cross-linking of CD3 and CD4 by anti-mouse goat serum an increased intracellular calcium signaling was obtained if MAX.16H5 incubation was performed for a maximum of 5 min (34). For longer incubation periods, the calcium signal decreased again indicating that full T cell activation by CD3 occurs rapidly within a short time (34). These data indicate a strong dependence of preincubation time. Furthermore, the increased calcium release only after cross-linking of CD3 and CD4 leads to the following speculation: the T cell activation was impaired due to transient but asynchronous activity of different kinases in T cells and intercellular cross-talk between T cells and monocytes was required (34).

At the time the studies were conducted, regulatory T cells (T_regs_) were not yet identified as important targets to follow in clinical GVHD research: By 2000, T_regs_ were identified as suppressors of autoimmunity *in vitro* and in mouse models [reviewed in Shevach (35) and Sakaguchi (36)]. However, the flow cytometric identification of T_regs_ remained difficult until they were specified as positive for CD4, CD25, and forkhead box protein P3 (FoxP3) in 2003 (37–39). Therefore, the clinical data on MAX.16H5 IgG_1_ lack information on the T_reg_ population.

#### Effects on B-Cell Crosstalk With T-Helper Cells and Immunoglobulin Secretion

PBMCs of healthy individuals were assessed for effects of MAX.16H5 IgG_1_ incubation on B-cell differentiation and resulting IgG and IgM production (40). It was found that incubation with MAX.16H5 IgG_1_ inhibited B-cell differentiation and following immunoglobulin (Ig) production (40). Even in the presence of mitogens and IL-2 or IL-4, MAX.16H5 IgG_1_ addition reduced Ig secretion (40). Moreover, the production of IL-2 and IL-4 by T-helper (T_h_) cells was minimally influenced by MAX.16H5 IgG_1_ under various stimulating conditions (40). Thus, cytokines were not responsible for lower Ig secretion after MAX.16H5 incubation. More likely, the reduction of direct cellular contacts between T_h_ and B cells by MAX.16H5 IgG_1_ and its F(ab')_2_ lead to reduced crosstalk between the two cell types causing reduced Ig secretion indicating that CD4-blockade by MAX.16H5 interferes with early T-B cell collaboration (40). In RA patients, Ig reduction was observed after MAX.16H5 IgG_1_ treatment, especially rheumatoid factor (RF) production, indicating that this effect is also present *in vivo* (13).

#### Effects on Monocytes

In the treatment of RA with MAX.16H5 IgG_1_, CD14^+^ monocytes in the PB were reduced one hour after infusion of MAX.16H5 IgG_1_ (13). Continuing the MAX.16H5 IgG_1_ treatment kept monocyte levels in normal ranges (13). The authors offer two possible explanations: either the monocyte/macrophage system is responsible for the depletion of antibody-coated T cells or the MAX.16H5 IgG_1_ bound to the CD4 molecule present on a monocyte subset results in temporary monocyte reduction in the PB (13). On the other hand, reduced crosstalk between T_h_ cells and monocytes may play a role in the observed reduction of monocyte activation (13). In different studies, the same RA patients were monitored for monocyte activation indicated by heightened neopterin serum values, MHC class II expression, monocyte counts, and IL-1 production prior to MAX.16H5 IgG_1_ application (14). These parameters could be reduced after MAX.16H5 IgG_1_ treatment (14). Moreover, elevated levels of soluble CD14 (sCD14) detected in five patients prior to MAX.16H5 IgG_1_ treatment were reduced in three patients after antibody application (18). IL-1 and IL-6 serum levels correlated to sCD14 concentrations in RA patients (18). A comparison between therapy responders and non-responders revealed reduced monocyte and T_h_ cell counts in the responder group, whereas both values increased again in the non-responder group after 1 week (18).

### Cytokine Release

In chronic inflammatory diseases such as RA and SLE, the release of pro-inflammatory cytokines plays a crucial role in disease progression. Elevated levels of cytokines produced by CD4^+^ cells including tumor necrosis factor (TNF)-α, IL-1, IL-6, and IL-17 favor disease pathogenesis [reviewed in Lourenço and La Cava (41) and McInnes and Schett (42)]. Monoclonal antibodies (mAbs) against these molecules or their respective receptors are therapeutic options to treat patients with autoimmune diseases (43). Therefore, the effect of the treatment with MAX.16H5 IgG_1_ targeting human CD4^+^ cells regarding cytokine release was studied in detail *in vivo* and *in vitro*.

IL-6 is known as the most important inducer and regulator of acute-phase response (44). Elevated IL-6 levels were measured in most RA patients before MAX.16H5 treatment (18). The IL-6 levels rapidly declined in four patients during the treatment course, which was observed in parallel with substantial clinical and laboratory improvement (18). On the other hand, one patient showed a slight increase of IL-6 during first treatment course and did not respond to treatment (18). One individual demonstrated a considerable increase of IL-6 and underwent an allergic skin reaction after the first injection (18). In that special patient, the IL-6 levels decreased to the pretreatment values after the treatment was stopped (18).

Besides the positive effect of IL-6 reduction, cytokine release due to MAX.16H5 IgG_1_ application was analyzed as potential side effect. During the therapy of RA patients with MAX.16H5 IgG_1_, symptomatic patients showed elevated serum levels of TNF-α, IFN-γ, and/or IL-2 (15). Comparison of the modulation efficacy of CD4^+^ T cells induced by MAX.16H5 treatment did not reveal any difference between patients without clinical adverse effects and those developing systemic side effects (15). The authors hypothesized that the clinical adverse effects were likely a result of lymphocyte activation and/or a monocyte/macrophage interaction with lymphocytes (15). A comparable side effect profile was described for mAb OKT3 treatment (45, 46) but MAX.16H5 IgG_1_ induced effects were milder and of much shorter duration which made a further treatment of the patient unnecessary (15). It has to be noted, that only a small cohort of patients systemically received MAX.16H5 IgG_1_ therapy for autoimmune disease treatment, which complicates drawing solid conclusions.

The cytokine production in SLE patients was analyzed *in vitro* as well (47). Spontaneous IL-6 secretion was heightened in blood cell cultures from patients with active SLE compared to cultures from inactive SLE patients and healthy controls (47). After incubation with MAX.16H5 IgG_1_, cell cultures of active SLE patients demonstrated reduced IL-6 levels, whereas TNF-α levels were not significantly altered (47). When samples from healthy volunteers were stimulated either with phytohemagglutinin (PHA) or lipopolysaccharide (LPS) MAX.16H5 IgG_1_ induced IL-6 decrease was found to be antibody dose-dependent (47). In control wells it was shown that the addition of methylprednisolone to the cell cultures of stimulated healthy volunteer samples, stimulated inactive SLE samples and unstimulated active SLE samples not only reduced IL-6 but also TNF-α secretion markedly (47). Summarized it was shown that MAX.16H5 antibody incubation altered stimulated IL-6 secretion of *in vitro* blood cell cultures obtained from SLE patients and healthy individuals. The decrease of stimulated IL-6 secretion was dose-dependent. Other than methylprednisolone MAX.16H5 IgG_1_ incubation did not influence TNF-α levels in these assays (47).

### Laboratory and Clinical Parameters

In contrast to other anti-CD4 antibodies, MAX.16H5 was the only one improving not only clinical but also laboratory parameters in RA patients [reviewed in Burmester and Emmrich (48)]. Additionally, MAX.16H5 application showed an effect on parameters which were associated with monocyte/macrophage activation (14). In general, a significant decrease of laboratory [erythrocyte sedimentation rate (ESR), RF titer, C-reactive protein (CRP) levels] and clinical parameters (Ritchie articular index and swollen joints) was observed (13). In one of the patients, no impact on ESR and CRP levels was observed during the first cycle of MAX.16H5 infusion (17). Four years before the treatment with MAX.16H5 IgG_1_, the patient was diagnosed with RA. Due to a trauma, he underwent splenectomy earlier in life (17). After the second course of MAX.16H5 therapy, ESR and CRP levels were reduced, possibly followed after decreased IL-6 serum values (17). Since the change in laboratory variables did not translate into an improvement of clinical parameters, low dose chlorambucil was implemented into the treatment regimen. The combination of CD4 directed antibody therapy together with chemotherapeutic medication resulted in clinical improvements which also translated in continued reduced levels of certain inflammatory parameters (ESR and CRP) (17). Overall, no adverse effects (especially infections) were observed (17).

The clinical parameters of two children were assessed in another study where MAX.16H5 was given *i.v*. for treatment of refractory juvenile chronic arthritis (JCA) (21). One patient benefited from the antibody therapy clinically within 1 week after the first antibody application. A second antibody application showed an even more improved response compared to the first course of treatment and symptoms like fever and rash were reduced for around 2 months (21). In the second patient, two cycles of treatment were needed to obtain notable improvement of clinical symptoms. Also, these juvenile patients did not show any signs of adverse side effects caused by the MAX.16H5 IgG_1_ antibody treatment (21).

Patients suffering from severe acute rejection after kidney transplantation also benefitted from the therapy with MAX.16H5 IgG_1_ (22). Histological signs of acute rejection (if present) disappeared as a response to the MAX.16H5 IgG_1_ treatment. All patients showed rapid decreasing serum creatinine levels within the first 3 days post injection. However, graft function was impaired in two patients 3–4 weeks after therapy and one patient experienced transplant rejection again after 10 weeks (22). The authors observed a rapid effect of the MAX.16H5 antibody in the treatment of acute rejection after kidney transplantation and concluded that CD4^+^ T cells seem to play an important role in the rejection process. They further suggested to implement the antibody therapy in established immunosuppression treatment protocols to improve therapeutic efficacy (22).

MAX.16H5 IgG_1_ application was also shown to be effective in the treatment of inflammatory bowel disease (IBD) (20). Especially, when other treatment options are exhausted and conventional therapeutics are ineffective MAX.16H5 IgG_1_ can be used as a treatment option in IBD (20). As also discussed in the treatment of autoimmune diseases earlier, single cycle administration of MAX.16H5 was insufficient to reach persistent therapeutic success (20).

### Other Effects

Since the exact mechanism of the MAX.16H5 IgG_1_ induced effects were not sufficiently explained neither *in vitro* nor *in vivo*, researchers focused on the intracellular signaling after antibody binding to its antigen. By using U937 target cells, the activation of complex inositol polyphosphate responses and Ca^2+^ increase after MAX.16H5 IgG_1_ antibody treatment was investigated *in vitro* independently from TCR signaling (49). The authors showed, that MAX.16H5 IgG_1_ incubation alone was not sufficient to induce Ca^2+^ increase in CD4-expressing cells (PB-monocytes and the monocyte cell line U937) (49). When goat anti-mouse antiserum was added, clear crosslinking of MAX.16H5 IgG_1_ was obtained leading to heightened Ca^2+^ levels (49). The outcome of experiments in U937 cells using F(ab)_2_ fragments of MAX.16H5 together with F(ab)_2_ crosslinking agents were not applicable to observations made with whole antibodies (49). The authors concluded that in U937 cells only “[…] crosslinking of CD4 and FcγR, but not cross-linking of CD4 alone specifically activates the inositol polyphosphate/Ca^2+^ signal transduction pathway” (49).

### Pharmacokinetics and Pharmacodynamics in Humans

Pharmacokinetic and pharmacodynamic studies with ^99m^Tc-labeled MAX.16H5 IgG_1_ were performed in RA patients (7, 11, 23, 24). In one study, patients received either *i.v*. injection of ^99m^Tc-labeled MAX.16H5 IgG_1_ antibody or *ex vivo* ^99m^Tc-labeled MAX.16H5 IgG_1_ incubated PB-lymphocytes (7). Following images/scans were taken using a gamma camera (7). The use of the CD4-directed MAX.16H5 IgG_1_ antibody for medical imaging in order to obtain information about disease progression in RA or also for diagnosis was promising since techniques used at that time, e.g., ^99m^Tc-early methylene diphosphonate (MDP) bone scans, were rather unsatisfactory (7). The authors concluded that ^99m^Tc-labeled MAX.16H5 imaged the localization of disease joints more precisely than MDP scans, which made it a promising tool for the diagnosis of autoimmune arthritis (7).

#### Organ Activity Distribution and Kinetics

In context with their participation in a therapeutic trial for RA [preliminary data after 2 patients enrolled evaluated in (50)], 4 patients received either radio-labeled MAX.16.H5 IgG_1_ antibody *i.v*. or PB-lymphocytes labeled with the antibody [1 patient] (11). The “[…] study was mainly concerned with the evaluation of the kinetic behavior of the antibody-labeled cells in the patients” (11). In general, the maximum of activity [100%] of the antibody or the antibody-labeled lymphocytes was reached within a few minutes in the heart and lung (ca. 4.5 min), whereas the maximum of radio-labeled activity was obtained after ca. 12 min in the spleen and about 19 min in the liver (11). However, 90 min after the injection, radiolabeled MAX.16H5 antibody activity reincreased in the patients' hip joints after a first injection peak (11). Interestingly, organ kinetic curves were comparable between patients receiving ^99m^Tc-labeled MAX.16H5 IgG_1_ and the patient who received *ex vivo* antibody-labeled lymphocytes (11). Additionally, study examined the whole-body radioactivity distribution at two different time points [4 and 24 h p.i.] (11). “The splenic uptake decreased by about 39% from 4 h […] to 24 h p.i. […]” (11). A moderate increase of activity measured in the liver was recorded (11). Nevertheless, at both time points, approximately 50% of radioactivity was measured in the bone marrow. Joints overall showed a rather low activity with 0.5 ± 0.09% for not-diseased joints vs. 2 (after 4 h) to 2.5% (after 24 h) for a single affected joint (11).

Another study (23) evaluated the kinetic differences between labeled MAX.16H5 IgG_1_ and polyclonal human immunoglobulin (HIG) in RA patients or healthy controls to exclude non-specific accumulation of immunoglobulin. Compared to HIG, ^99m^Tc-labeled MAX.16H5 IgG_1_ showed a higher uptake in the liver and in the spleen of RA patients at 24 h p.i. (23). Since the MAX.16H5 IgG_1_ “[…] showed a higher target-to-background ratio in arthritic knee and elbow joints in comparison to polyclonal HIG used for conventional imaging […]” the authors discussed a potential beneficial application of the antibody in the “[…] detection of inflammatory infiltrates rich in CD4-positive cells” (23).

### Adverse Effects

The adverse effects observed in studies with human patients or healthy volunteers are summarized in Table 1. In different trials using the ^99m^Tc-labeled MAX.16H5 IgG_1_, no adverse reaction was observed after the intravenous application (7, 11, 23, 24).

Several studies examined the treatment of RA patients with MAX.16H5 IgG_1_. In general, only occasional and minor side effects were observed which probably resulted from short lived cytokine peaks (15). The infusions were well-tolerated [reviewed in Burmester and Emmrich (48)]. Immediate adverse effects were allergic reactions on rare occasion as well as nausea and fever being symptomatic for the development of a mild to moderate cytokine release syndrome (15). A long-term effect on the topic of laboratory parameters was the development of HAMAs (13, 16, 21). Approximately 25% of the HAMA activity was directed against idiotypic determinants (16). Significant HAMA concentrations were measured between 2 and 12 weeks p.i. (16). Still, in contrast to monoclonal antibodies directed against other T cell epitopes, the amounts of these antibodies were low and never exceeded 2.0 (after 1 cycle) or 2.2 mg/l (after 2 cycles) (16). Thus, patients could be retreated without loss of efficacy (13) as similarly shown with other anti-human CD4 antibodies (51).

The reduced CD4^+^ T cell numbers did not result in infectious problems in any study (48). Together with an unaltered or even elevated T cell reactivity *in vitro* [4 patients showed heightened T cell reactivity to common antigens and mitogens when CD4^+^ cell numbers were still reduced (13)], this observation points to low numbers of CD4^+^ T_h_ cells being sufficient to maintain the function of the cellular immune system (13, 48).

In the trial using MAX.16H5 IgG_1_ in the treatment of refractory JCA in two patients the first treatment course was tolerated without side effects (21). Application of MAX.16H5 IgG_1_ in the treatment of either SLE or IBD patients did not show any side or adverse effect (12, 20). In the therapy of acute rejection in long-term renal allograft recipients, no adverse effects due to the treatment were mentioned in the original article (22).

Although not all trials showed the development of HAMAs as side effect, the possibility of developing immune reactions against the murine IgG_1_ Fc-part remains. This risk was considered to be reduced by the development of a chimerized, humanized version of the MAX.16H5 IgG_4_ antibody.

## Development of a Chimerized MAX.16H5 IgG_4_ Monoclonal Antibody

**Figure 1:**
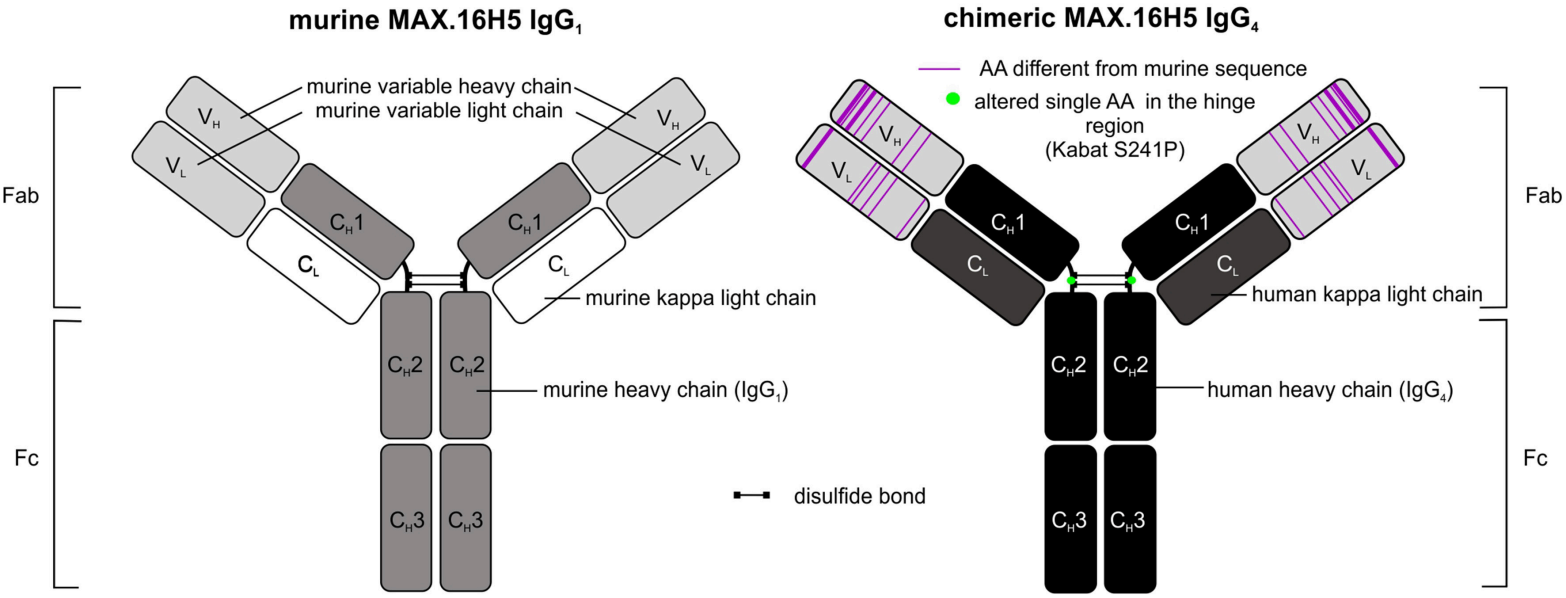
Schematic representation of murine MAX.16H5 IgG_1_ and chimeric MAX.16H5 IgG_4_ antibodies.

The use of murine antibody formats for therapeutic interventions was shown to be connected to the development of several side effects, e.g., antibody responses (HAMA) or allergic reactions, which led to the development of optimization protocols in antibody design using recombinant DNA tools already in the 1980's (52, 53). Morrison et al. described a process called “chimerization” where heavy and light chain variable DNA sequences of a murine antibody were connected to DNA sequences encoding human IgG_1/2_ and sequences encoding for the human kappa light chain, respectively (53). Morrison and colleagues discussed the potential of such “near-human” antibody formats with respect to reduced side effect profiles when administered *in vivo* (53). In 1997, the approval of rituximab, a chimeric CD20-directed IgG_1_ antibody for the treatment of lymphoma eventually paved the way for modified antibody formats (54). In December 2018, 75 antibodies as therapeutics were approved for the treatment of a variety of diseases including 9 antibodies carrying human Fc domains and murine variable sequence motifs and are therefore defined as chimeric (43). The aforementioned CD20-directed antibody rituximab is listed as well as e.g., obiltoxaximab, which reached US-approval more recently (2016) and is used for the treatment and prophylaxis of inhalational anthrax (55). The chimerization of MAX.16H5 was promoted in order to reduce immunogenicity of the antibody for potential clinical applications. The MAX.16H5 chimerization process was started in 2007. A CD4-directed murine IgG_1_ antibody-expressing hybridoma clone was used as starting material. By combining cloning and sequencing techniques together with *in silico* modeling, variable regions of light and heavy chains were extracted, analyzed, modified and connected to human constant regions as commissioned (Figure 1) (56). Mammalian cells were used for the production of the chimeric antibody (56). Binding profiles of MAX.16H5 IgG_4_ were comparable with the murine MAX.16H5 IgG_1_ proving that the correct variable regions were chosen to generate the chimeric antibody (56).

In general, Fc parts of antibodies are known to mediate effector mechanisms which include their interaction with both certain Fc receptors expressed on effector cells and the activation of the complement system (57). Depending on their Fc-binding capacities and mediated effector mechanisms, therapeutic antibodies can be used for different therapeutic purposes. In the setting of HSCT or GVHD, an Fc-mediated depletion of CD4^+^ cells was not our desired therapeutic approach. Therefore, MAX.16H5 variable domains were specifically connected to human IgG_4_ and not to human IgG_1_ constant domains since it is known that IgG_4_ is a weak activator of ADCC and CDC (58) [reviewed in Davies and Sutton (59)]. Because of the functional features of the IgG_4_ isotype, its translation into the clinical application in the field of immune checkpoint blockade was quite successful. To date, pembrolizumab (MK-3475) and nivolumab (MDX-1106), two antibodies harboring IgG_4_ backbones, are approved for immune checkpoint blockade in the USA and the EU (43). The molecules effectively inhibited programmed cell death protein 1/programmed cell death 1 ligand 1 (PD-1/PD-L1) interactions but were shown to be inactive eliciting Fc-mediated effector functions [reviewed in Topalian et al. (60)]. Another challenge in the development of IgG_4_-based therapeutic antibodies was the isotype's feature of the so-called Fab-arm exchange where single heavy chain-light chain dimers can form bivalent antibodies with other single heavy chain-light chain dimers (61). From a biological point of view it was discussed that the bivalency of IgG_4_ molecules may reduce their “pathological potential” (61). For certain applications, therapeutic antibody manufacturing can be challenging due to the Fab-arm exchange of IgG_4_. This led to the development of several antibody structure optimization strategies in the past (59, 61). A study published in 2009 showed that bispecific IgG_4_ antibodies were detectable in blood samples from patients who received an unmodified IgG_4_-based therapeutic antibody (62). The antibody formed half-molecules *in vivo* and furthermore, assembled to bispecific antibodies with patient-specific endogenous IgG_4_ (62). The group introduced a S228P amino acid substitution in the hinge-region of the antibody and showed the prevention of Fab-arm exchange impressively (62). The MAX.16H5 IgG_4_ sequence was optimized in the same manner in order to prevent Fab-arm exchange (Figure 1) (56), to ensure the stability of the antibody, and the reliability of the manufacturing process during clinical development and GMP production.

## Non-clinical Development of the Chimerized Anti-human Anti-CD4 Antibody—Studies of MAX.16H5 IgG_1_ and IgG_4_ in Murine GVHD Models

It was sought to examine the properties of the antibody's influence on the immune system using mouse models. To study the effect of the MAX.16H5 antibody against human CD4 expressing cells in mice, a genetically modified murine model with a C56BL/6 background was developed, which functionally expressed human CD4 on T cells while the murine CD4 gene was knocked-out (63–65). Additionally to the murine MHC II the T cells expressed human HLA-DR17 (63–65). In first experiments, spleen and lymph node samples of triple transgenic (TTG) mice previously immunized with tetanus toxoid (TT) showed reduced immune response after MAX.16H5 IgG_1_ treatment and re-stimulation with TT *ex vivo* (66). The same effect emerged *in vivo* after injection with 15 μg/g body weight (BW) MAX.16H5 IgG_1_ (67) and the F(ab)_2_ of MAX.16H5 was as potent as the whole antibody (65). Surprisingly, unresponsiveness was preserved after the mice underwent another antigen boost without prior antibody administration, thus indicating a long-lasting but not depleting effect of MAX.16H5, which was moreover antigen-specific and dependent on the ability to form the immunological synapse between CD4 and HLA-DR (65). Therefore, we speculate, that MAX.16H5 does not induce a general immune suppression but only to antigens present simultaneously with or shortly after antibody treatment, most likely while the antibody is still bound to its ligand when the HLA-DR molecule encounters the TCR. This observation led to the idea that MAX.16H5 may conquer an old challenge in immunology: induction of specific tolerance and influencing both host-vs.-graft and graft-vs.-host reactions. The latter severely limit the application of allogeneic hematopoietic stem cell (HSC) and immune cell transplantations for the treatment of, e.g., autoimmune diseases or hematopoietic cancers such as leukemia.

Acute GVHD is the main complication of allogeneic hematopoietic stem cell transplantation (HSCT) and the main reason for early transplantation-associated mortality (68). Conventional immunosuppressive drugs such as corticosteroids are not specific and suppress the entire immune system (69). So far, no therapy manages HSCT or donor lymphocyte infusion without the need for conventional systemic immunosuppressive drugs. Promising *in vivo* data were obtained regarding the feasibility and effectivity of *ex vivo* graft incubation with MAX.16H5 IgG_1_ and the antibody's influence on GVHD down modulation after allogeneic full-mismatch immune stem cell transplantation if the graft from TTG mice was preincubated with the antibody (70–72). Of note, removing unbound antibody molecules from the graft did not reduce its effectiveness (70–72). The graft's unresponsiveness to allogeneic BALB/c^wt^ antigens was even preserved if immune and stem cells from transplanted GVHD-free mice were transferred to new BALB/c^wt^ mice without MAX.16H5 preincubation—a phenomenon called “infectious tolerance” (71). This phenomenon is possibly achieved by heightened levels of T_regs_ present in mice receiving MAX.16H5-preincubated grafts suggesting a possible role for long-term unresponsiveness *in vivo* (71). Two studies did not only focus on accelerated GVHD but also on the maintained GVL effect mediated by the transplanted antibody incubated immune cell graft. To investigate the GVL effect mediated by the transplanted graft, BALB/c^wt^ animals did not only receive TTG immune cell grafts but also P815 cells, a murine mastocytoma cell line (70). It was shown that graft preincubation with the murine MAX.16H5 IgG_1_ antibody did not influence the GVL effect which was induced by the transplanted immune cells (70, 71). In another murine model, the murine MAX.16H5 IgG_1_ also prolonged the survival of recipient C3H/HeN mice (receiving a TTG immune cell graft to induce GVHD) even if they were co-transplanted with myeloblast-like murine cell line 32D Clone 3 (32D) expressing human Fms like tyrosine kinase 3 with the internal tandem repeat duplication (FLT3^ITD^), which constitutes an aggressive acute myeloid leukemia (AML) cell line model (72).

The *ex vivo* graft incubation with MAX.16H5 together with a subsequent washing protocol was found to be an attractive and promising therapeutic setting in HSCT (31, 70, 71). It got even more into focus when patients, who received a therapeutic antibody, suffered from side-effects which were mainly caused by a systemic inflammatory response as a response to systemic application (73). In 2006, unpredicted side-effects emerged during a phase-I (first-in-man) clinical trial with a CD28-directed monoclonal antibody called TGN1412 (73, 74). The antibody was developed to modulate T_reg_ expansion and was praised as “a promising novel tool for the treatment of human autoimmune diseases” (75). As of today, the cytokine release syndrome is a known side-effect of different immunotherapeutic interventions such as therapeutic antibodies (e.g., CD20-directed mAbs, bispecific T cell engagers, and immune checkpoint inhibitors) or chimeric antigen receptor (CAR) T cells [reviewed in Shimabukuro-Vornhagen et al. (76)]. Due to safety considerations, the development and optimization of the MAX.16H5 antibody format for the treatment of GVHD was shifted to *ex vivo* graft incubation rather than systemic administration of the therapeutic antibody.

After the chimerized MAX.16H5 IgG_4_ was available, its effectiveness in preventing GVHD was evaluated using a NOD.Cg-*Prkdc*^*scid*^ *Il2rg*^*tm*1*Wjl*^/SzJ (NSG) mouse model (31). Here, mice received a xenogeneic human PBMC transplant to initiate acute GVHD (31). Both variants, the murine IgG_1_ and the chimeric IgG_4_ of the MAX.16H5 antibody, were compared regarding their ability to down regulate GVHD development. Unspecific isotype antibodies were used as controls in these experiments (31). The data were published in 2016 and showed that both the murine IgG_1_ and the chimerized IgG_4_ were able to prevent GVHD in this experimental setting (31). The mice received immune cell grafts which were *ex vivo* incubated with the respective antibodies making a systemic application of MAX.16H5 unnecessary. After 2 h of MAX.16H5 IgG_1_ or -IgG_4_ preincubation, the grafts were washed with PBS to remove excess, unbound antibodies. Finally 2 × 10^7^ MAX.16H5 or isotype control treated graft cells were diluted in 150 μl 0.9% NaCl and were injected into the tail vein of the animals (31). Mice receiving MAX.16H5 murine IgG_1_ or chimeric IgG_4_ antibody incubated grafts showed a significant prolonged survival in comparison to mice receiving grafts incubated with isotype control antibodies (31). During these animal experiments, data were obtained collecting several parameters such as general health status (e.g., fur, weight, behavior, mobility), immune cell reconstitution (white blood cell counts, flow cytometric analyses of human T cell, B cell, and lymphocyte markers) and histological data (apoptotic cells in the gut of the mice, TUNEL) (31). Importantly, MAX.16H5 IgG_1_ (murine) or IgG_4_ (chimeric) incubation of immune cell grafts comparably weakened GVHD development significantly but did not impair the engraftment of the transplanted cells (31).

## Concluding Remarks

It was early known that even the murine IgG_1_ istoype of MAX.16H5 does not stimulate CDC or ADCC (13), but the mechanism of action is still not understood. Over the years of development, many theories have been arosen on its mechanism of action to induce tolerance. Despite being of interest, most data were not published as they showed negative results. Due to the quick responses observed in clinical trials, it was speculated that the antibody's mechanism was independent of antigen recognition which would take longer but was more likely caused by inhibition of preactivated IL-2-dependent T cells (32). However, this theory cannot explain why the antibody is effective in GVHD prevention after an *ex vivo* incubation, i.e., before the cells of the transplant had antigen contact. Moreover, the monocyte/macrophage system was thought to be involved, but this idea too was in conflict with reported data (13). Rising T_reg_ amounts in a murine model (71) are probably a mediator of tolerance but do not explain the mechanism how tolerance is achieved.

**Figure 2:**
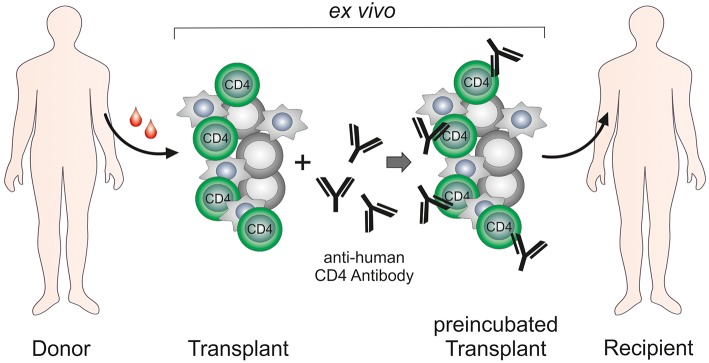
*Ex vivo* treatment of hematopoietic stem cell/immune cell grafts by anti-human CD4 antibody MAX.16H5.

The majority of the studies performed with the anti-human CD4 antibody MAX.16H5 focused on the treatment of autoimmune diseases such as RA, SLE, IDB and JCA and revealed striking results that identified the MAX.16H5 antibody as a promising alternative for conventional therapeutics. Following initial systemic application of MAX.16H5, a new strategy was developed leading to similar success in therapy and improved safety of patients: graft CD4^+^ cells were incubated *ex vivo* with the MAX.16H5 antibody and re-infused into the patient (Figure 2). This innovative approach extended the scope from the treatment of autoimmune diseases to hematological malignancies. By *ex vivo* incubation of an allogeneic immune- and stem cell transplant with the epitope-specific anti-human CD4 antibody MAX.16H5, a new therapy strategy has emerged for the first time enabling both the preservation of the GVL effect of the transplant and the permanent suppression of GVHD without the need for conventional immunosuppression. The following essential benefits of this innovative therapeutic approach can be expected: First, the treatment of a human immune cell transplant does not deteriorate the anti-tumor effectiveness with regard to different leukemia types. Second, it is expected that an allogeneic HSC graft treated with MAX.16H5 anti-human CD4 antibodies leads to a general improvement of the survival due to suppression of the GVHD. Furthermore, the dosage and amount of conventional immunosuppressive drugs (toxicity, side effects, and duration of the treatment) can be reduced and the patients' quality of life could be improved. Due to the suppression of the GVHD, patients lacking a suitable donor will be applicable to receive donor cells whose transplantation normally would be associated with a higher risk for GVHD (e.g., more HLA mismatches). Thus, immune cell therapies will become applicable to cure other diseases (e.g., autoimmune diseases and primary immunodeficiencies) whose curative treatment regimen does currently not include this form of therapy because of a high risk for GVHD development. Finally yet importantly, the incubation of the allogeneic HSCT grafts with the epitope-specific anti-human CD4 antibody MAX.16H5 can be performed outside of the body which reduces side effects and therapy costs, antibody amounts as well as improves the safety of the transplantation remarkably.

## Author Contributions

LS, AD, NH, UT, and SF analyzed the publications, created the figures and tables, and wrote the paper.

### Conflict of Interest Statement

The authors declare that the research was conducted in the absence of any commercial or financial relationships that could be construed as a potential conflict of interest.

## Abbreviations

AA: amino acid
ADCC: antibody dependent cellular cytotoxicity
aGVHD: acute graft-vs.-host disease
AML: acute myeloid leukemia
BW: body weight
CDC: complement dependent cytolysis
CEA: carcinoembryonic antigen
CRP: C-reactive protein
ELISA: enzyme-linked immunosorbent assay
ESR: erythrocyte sedimentation rate
F(ab)_2_: fragment antigen binding region
Fc: fragment crystallizable region
FcR: Fc receptor
FLT3: Fms like tyrosine kinase 3
FoxP3: forkhead box protein P3
GVHD: graft-vs.-host disease
GVL effect: graft-vs.-leukemia effect
HAMA: human anti-mouse antibody
HIV: human immunodeficiency virus
HLA: human leukocyte antigen
HSC: hematopoietic stem cell
HSCT: hematopoietic stem cell transplantation
IBD: inflammatory bowel disease
Ig: immunoglobulin
IL: interleukin
ITD: internal tandem duplication
ITAM: immunoreceptor tyrosine-based activating motif
ITIM: immunoreceptor tyrosine-based inhibitory motif
i.v.: intravenous
JCA: juvenile chronic arthritis
Lck: lymphocyte-specific protein tyrosine kinase
mAb: monoclonal antibody
LPS: lipopolysaccharide
MHC: major histocompatibility complex
NSG: NOD.Cg-*Prkdc*^*scid*^ *Il2rg*^*tm*1*Wjl*^/SzJ
PB: peripheral blood
PBMC: peripheral blood mononuclear cell
PD-1: programmed cell death protein 1
PD-L1: programmed cell death 1 ligand 1
PHA: phytohemagglutinin
p.i.: post injection
RA: rheumatoid arthritis
RF: rheumatoid factor
sCD14: soluble CD14
SLE: systemic lupus erythematosus
TCR: T-cell receptor
T_h_ cell: T-helper cell
T_reg_: regulatory T cell
TT: tetanus toxoid.
